# Population aging and corporate tax avoidance: Suppression or promotion?

**DOI:** 10.1371/journal.pone.0316211

**Published:** 2025-08-11

**Authors:** Luzhuang Qi, Peng Liang

**Affiliations:** 1 School of Economics and Management, Shihezi University, Shihezi, China; 2 School of Accounting, Capital University of Economics and Business, Beijing, China; An-Najah National University, PALESTINE, STATE OF

## Abstract

Addressing population aging has emerged as a paramount national strategic priority. Against the backdrop of a continual rise in population aging and considerable escalation in labor costs within China, this study aims to investigate whether firms, confronted with the burden of labor costs, intensify their tax avoidance motives to generate a “stimulating effect” or whether they are compelled to adapt traditional factors, thereby reducing tax avoidance and exerting a “restraining effect”. To address this inquiry, we empirically examine the impact of population aging on corporate tax avoidance and its underlying mechanisms utilizing a sample of non-financial listed companies in China’s A-share market spanning from 2008 to 2023. Our findings substantiate that the level of population aging significantly diminishes firms’ tax avoidance motives, confirming the presence of a “restraining effect”. Mechanism tests unveil that population aging, through capital-labor substitution, fosters research and development innovation and improves production efficiency, thus curbing firms’ tax avoidance behavior and affirming the existence of the “factor substitution effect” “innovation capacity effect” and “resource allocation effect”. Heterogeneity analysis reveals that the inhibitory impact of population aging on corporate tax avoidance is more pronounced in labor-intensive industries, entities with limited financing capacity, weak financial conditions, and adverse external financial conditions. By examining the economic ramifications of population aging from a micro-level perspective on corporate financial decision-making and enriching the existing literature on corporate tax avoidance through a population economics lens, this study provides valuable insights for firms undergoing transformation and informs policy formulation in response to the challenges posed by population aging.

## 1. Introduction

Population is one of the most important variables in the economy. In recent years, aging has gradually become a trend of the world’s population, and aging is no longer found only in developed countries; more and more developing countries are also entering the aging stage. “Implementing the National Strategy for Actively Responding to Population Aging” has been upgraded to a national strategy in 2020, fully demonstrating the seriousness of the problem of population aging in China and the great importance attached to it by the Chinese government. According to the Seventh National Population Census Bulletin of 2021, the population of individuals aged 60 and above in China has reached 264 million, accounting for 18.70% of the total population. Moreover, the proportion of individuals aged 65 and above stands at 13.50%, approaching the threshold of moderate aging. Compared to the Sixth National Population Census in 2010, the share of individuals aged 60 and above has increased by 5.44 percentage points, indicating an acceleration in the pace of population aging compared to the less than 3 percentage points increase observed between 2000 and 2010. Projections from the United Nations’ World Population Prospects reveal that China’s old-age dependency ratio is expected to rise by 10.5 percentage points in the next decade.

Population aging not only precipitates a decrease in the potential economic growth rate from the supply side but also engenders persistent deficiencies in aggregate demand, potentially leading to long-term unemployment. Consequently, effectively addressing the adverse repercussions of population aging has emerged as a critical concern in both theoretical and practical realms for the government and the academic community. Simultaneously, human capital has undergone profound changes alongside population aging. Population aging reduces the supply of labor, prompting enterprises to substitute capital for labor. Population aging enhances the quality of enterprise labor supply, improving research and development (R&D) innovation capabilities and profitability. It also encourages enterprises to optimize resource allocation, thereby increasing total factor productivity (TFP) and fundamentally enhancing production efficiency and core competitiveness. These changes may reduce the need for enterprises to save cash flow and alleviate financing constraints through tax avoidance, thereby diminishing their motivation for tax evasion. This paper, adopting a population economics perspective, aims to explore the ramifications of population aging on corporate tax behavior and elucidate the underlying mechanisms within the context of a shrinking labor force and rising costs. This critical issue warrants further examination.

The examination of the economic ramifications of population aging through the lens of tax avoidance is warranted due to its significance as a crucial avenue and prevalent behavior adopted by firms to mitigate tax burdens and pursue value maximization. However, from a national perspective, such tax avoidance practices also result in a reduction of national tax revenue, leading to substantial losses in fiscal income for the nation [1]. Corporate income tax, as the second-largest source of tax revenue in China, accounted for 18.69% of national tax revenue in 2011 and increased to 26.22% in 2022. Its consistent growth holds immense importance for Chinese governments at all levels, aiding in the reduction of fiscal deficits and the improvement of public resource allocation. Nevertheless, for companies, corporate income tax represents a significant cash outflow, amounting to a quarter of pre-tax profits for firms following the implementation of the new corporate income tax law in 2008. In recent years, with mounting downward economic pressure in China and the disruptive impacts of events such as financial crises and the COVID-19 pandemic, numerous companies find themselves facing liquidity crises, motivating them to curtail cash outflows through tax avoidance activities [2–3]. Moreover, when engaging in tax avoidance strategies, companies often employ highly intricate and opaque transactional practices to conceal their behavior and evade detection by tax authorities. In companies with imperfect internal and external oversight mechanisms, these transactions are susceptible to becoming tools for insiders to engage in opportunistic behavior and reap personal gains, thereby severely compromising shareholder interests. Consequently, the reduction of corporate tax avoidance levels has become a matter of widespread concern within society.

In order to address the aforementioned research question, this study endeavors to establish a link between macro-level population data and micro-level firm data, specifically focusing on China’s A-share non-financial listed companies spanning the period from 2008 to 2023. The objective is to meticulously examine the impact of population aging on corporate tax avoidance and delve into the underlying mechanisms driving this relationship. The findings of this study demonstrate a substantial decrease in corporate tax avoidance as the extent of population aging intensifies. Robustness tests, encompassing alternative variable measurements, sample handling techniques, and endogeneity analysis, further reinforce the reliability of this outcome. The results obtained from the mechanism analysis shed light on the role of population aging in diminishing corporate tax avoidance by fostering capital substitution for labor, augmenting research and development innovation capabilities, and enhancing production efficiency. These findings offer empirical evidence supporting the existence of the “factor substitution effect” “innovation capacity effect” and “resource allocation effect”. Additionally, heterogeneity analysis uncovers that the inhibitory impact of population aging on corporate tax avoidance is more prominent in labor-intensive industries, weaker financing capacities, poorer financial conditions, and unfavorable external financial conditions.

This paper makes three significant contributions to the existing body of research. Firstly, this research expands the literature on the economic implications of population aging at the firm level. While some recent studies have examined the link between population aging and corporate innovation [4–5], Firms’ (non)responses [6] and marketing response [7] from a firm perspective, there remains a lack of comprehensive research on the economic consequences of population aging concerning corporate tax avoidance. This study expands and enriches the literature related to the study of the economic consequences of the demographic transition at the firm level, and can also provide some empirical references and lessons for government departments to carry out demographic policy formulation.

Secondly, this study contributes to the literature on factors influencing corporate tax avoidance by providing a unique macro-demographic perspective. Existing studies on the motives behind corporate tax avoidance have primarily relied on resource dependency theory, agency theory, and signaling theory. Population aging alters the labor resource composition of firms, profoundly influencing their decision-making processes. Furthermore, as a fundamental long-term variable, population aging may exhibit distinct mechanisms compared to short-term shocks such as financial crises. This paper supports the traditional tax avoidance perspective within the resource dependency theory framework, presents a fresh perspective on the factors influencing corporate tax avoidance and provides empirical evidence that aligns with traditional tax avoidance theories.

Thirdly, this paper discusses the heterogeneity of the three mechanisms of “factor substitution effect” “innovation capacity effect” and “resource allocation effect” and the internal and external environment of enterprises. It helps to further analyze the causal relationship and provides micro-level decision-making references for the internal factor adjustment of enterprises and the government to alleviate the negative impact of population aging.

## 2. Literature review

### 2.1 Study of the economic consequences of population aging

Population aging presents a myriad of social and economic challenges, encompassing a declining labor force [8,9], sharping income inequality [10–11], adversely affecting exports upgrading [12], increasing the burden of social pensions [13–14], slowing economic growth [8–9,15], and enhancing economic risks and challenges, among others, yielding significant adverse consequences that cannot be disregarded. Extensive scholarly research has focused on population aging, predominantly at macroeconomic and household levels, such as macroeconomics [16–19], housing consumption [20], labor markets [21], public health insurance [22–24], international trade [25], export upgrading [12], carbon emissions [26–29], environmental sustainability [30]. Nevertheless, limited attention has been devoted to micro-level analyses investigating the impact of population aging on firms’ (non) response [6] and proactive marketing response [7].

### 2.2 Research on corporate tax avoidance motivations

Tax avoidance represents a practice characterized by both significant potential returns and simultaneous risks. The decision-making process regarding whether and to what extent corporations engage in tax avoidance entails a comprehensive evaluation of the benefits, costs, and associated level of risk that they are willing to assume. Companies commonly employ diverse strategies, such as income concealment, expense inflation, and transfer pricing, as means to evade taxes, thereby augmenting their disposable cash flow [31], alleviating financing pressures, and potentially enhancing after-tax profits [32]. Nonetheless, tax avoidance carries substantial costs, encompassing planning and implementation expenses (in terms of managerial time and effort), potential penalties imposed by tax authorities, depreciation of the value of publicly traded firms, and the inherent risks linked to financial statement restatements [33–34]. Penalties resulting from tax authority detection of tax avoidance, coupled with the accompanying reputational damage [35], can reach approximately 40% of the anticipated tax avoidance benefits [36]. Non-tax costs stemming from tax avoidance may even exceed the actual tax benefits, encompassing increased agency costs [37], diminished capacity for debt financing, and heightened financing costs [38–39], elevated risks of stock price collapse [34], diminished investment efficiency [40], and higher audit fees [41], among others.

## 3. Theoretical analysis and research hypothesis

Population aging constitutes a fundamental structural transformation in the labor input of firms, and its impact on corporate tax avoidance can be examined through the lens of the traditional tax avoidance view within the resource dependence theory framework. This perspective encompasses three mechanisms: factor substitution effects, innovation capacity effects, and resource allocation effects, which collectively shape the motives driving corporate tax avoidance in response to the influence of population aging on firms’ labor resources.

Firstly, population aging reduces the supply of labor force, prompting enterprises to substitute capital for labor, reducing the corporate tax avoidance motivation, and exhibiting an input substitution effect. Population aging has altered the supply and demand conditions for corporate labor, increasing the difficulty of employee recruitment for businesses, as well as the direct labor costs associated with recruitment, training, and adjustment. Moreover, it has led to an increased social security burden on enterprises, augmenting their tax liabilities and tax avoidance incentives. According to factor substitution theory, capital and labor are substitutable production factors, and their utilization depends on relative prices and marginal productivity. The relative increase in labor costs inevitably incentivizes firms to employ machinery and other capital-intensive technologies as substitutes for labor [42]. Consequently, population aging compels firms to invest in fixed assets like intelligent systems and mechanized equipment, thereby increasing automation, intelligence, and digitization levels in production processes. By substituting labor with capital-intensive technologies such as industrial robots, firms can improve the marginal productivity of labor and attain a new equilibrium along the isoquant curve [43]. Whether driven by the pursuit of higher labor productivity or the pressures of rising labor costs, rational firms actively utilize advanced machinery to replace low-skilled workers, leading to technological progress and enhanced labor productivity [44]. Consequently, firms get rid of the labor cost burden by substituting capital for labor, reducing the incentive for tax avoidance.

Secondly, population aging can enhance a firm’s R&D innovation capabilities and profitability, while reducing the firm’s motivation for tax avoidance, exhibiting an innovation capability effect. On the one hand, education plays a vital role in enhancing human capital. Population aging leads to the miniaturization of family sizes, which is more conducive to the concentration of educational resources within households, promoting investment and accumulation of human capital, thereby enhancing the quality of labor supply. The enhancement of human capital, coupled with accumulated valuable skills over time, directly enhances firms’ product development and technological innovation capacities and indirectly enhances their R&D innovation capabilities through knowledge spillover effects, enabling the absorption and utilization of advanced knowledge and technologies [45]. Consequently, this drives improvements in firms’ production efficiency, profitability, and long-term growth [46]. On the other hand, aside from human capital accumulation and enhancement, improvements in labor quality encompass reduced employee turnover rates, increased effort, and the provision of valuable experience, loyalty, confidence, and morale within firms. These factors contribute to a deeper understanding of production environments and methods, facilitating enhancements in labor proficiency, technological innovation, organizational and production management, among others, thereby further improving firms’ technological innovation capacities. Thus, Population aging improves the quality of labor supply and R&D innovation, boosting corporate profitability and dampening the incentives for corporate tax avoidance.

Finally, population aging prompts enterprises to optimize resource allocation, enhancing TFP, thereby reducing tax avoidance motivation and exhibiting a resource allocation effect. TFP represents the overall efficiency of transforming various input combinations into output and fundamentally reflects resource allocation efficiency. Its improvement relies on factors such as technological progress, capital allocation efficiency, economies of scale, organizational and institutional innovations, and business environments. On the one hand, in the context of population aging, both governments and firms increasingly prioritize technological advancements and improved resource allocation efficiency, with the overall increase in innovation inputs providing firms with additional resources for innovation. The impact of population aging on labor quantity and quality compels firms to substitute capital for labor, such as employing industrial robots, and increase investments in R&D innovation. This shift in resource allocation towards sectors with higher productivity and greater potential enhances firms’ TFP [47]. On the other hand, the aging of the population leads to a reduction in the number of laborers and an increase in labor costs, prompting the optimization of the allocation of intangible factor resources such as the organizational system and the efficiency of resource allocation, which reduces managerial slack, improves managerial efficiency and productivity, and thus enhances the TFP of the enterprise [48]. Therefore, faced with the long-term trend of population aging, rational management teams inhibit short-sighted behavior and prioritize firms’ long-term interests. They strive to enhance TFP, ultimately improving production efficiency and core competitiveness, which in turn reduces the need for tax avoidance as a means of preserving cash flows and alleviating financing constraints.

The aforementioned analysis demonstrates that the aging population can diminish enterprises’ tax avoidance motives through the “factor substitution effect”, “innovation capacity effect”, and “resource allocation effect”. Consequently, this study proposes the research hypothesis H1:

H1: Population aging is expected to decrease corporate tax avoidance behavior.

## 4. Research design

### 4.1 Research sample

This study focuses on A-share listed companies in the Shanghai and Shenzhen stock markets during the period from 2008 to 2023. The selection of this sample period is based on two primary considerations. Firstly, in 2008, China implemented the new “Enterprise Income Tax Law”, which brought significant adjustments to the corporate income tax system. By setting the sample start year as 2008, it ensures consistency in the tax environment and comparability of data among companies. Secondly, starting from 2007, China adopted the new “Enterprise Accounting Standards”, which introduced various changes, including income tax information. Commencing the analysis from 2008 guarantees comparability of income tax data among companies. Data on population aging are sourced from the “China Population Statistics Yearbook (1988-2006)” and the “China Population and Employment Statistics Yearbook (2007-2023)”. Relevant data for listed companies are obtained from China Stock Market & Accounting Research Database (CSMAR), while nominal corporate income tax rate data are sourced from the Wind database. The sample selection process follows the following steps: (1) exclusion of financial sector samples, (2) exclusion of ST and PT listed companies, (3) exclusion of samples with negative net assets, (4) exclusion of samples with missing data, and (5) exclusion of abnormal observations with actual income tax rates below 0 or above 1. After the screening process, a total of 21,438 unbalanced panel data observations for firm-year combinations are obtained. To mitigate the impact of outliers on empirical results, all continuous variables are Winsorized by truncating extreme values at the 1% level.

### 4.2 Variable definitions

#### 4.2.1 Population aging.

This is a key variable examined in this study, specifically the population aging rate (*OLD*) in the province where the firm is located. According to the United Nations standard, a region is considered to have entered an aging society if the proportion of the population aged 60 and above reaches 10% or the proportion of the population aged 65 and above reaches 7%. In line with the research conducted by [49], this article adopts the proportion of the population aged 65 and above as the primary indicator of population aging. Additionally, we use the old-age dependency ratio (*OLD1*), which measures the ratio of the population aged 65 and above to the working-age population aged 15–64, in robustness tests to ensure the reliability of the findings.

#### 4.2.2 Corporate tax avoidance.

It encompasses various measures that can be classified into three main categories. The first category involves accounting-tax differences of enterprises and their variations. The second category focuses on the effective income tax rate of enterprises and its variations, while the third category examines the cash income tax rate and its variants. In line with previous literature [50], this study selects two commonly used indicators from the first category, which are accounting-tax differences (*BTD*) and accounting-tax differences excluding the effect of accrued profits (*DDBTD*), to measure corporate tax avoidance [50]. Additionally, as robustness tests, two measures from the second category, namely the difference between the nominal and effective income tax rates (*RATE*_*DIFF*) and the average of this difference over a five-year period (*LRATE*_*DIFF*), are employed. The *RATE*_*DIFF* is calculated as the ratio of pre-tax accounting profit minus taxable income to total assets at the end of the period, where taxable income is determined as (income tax expense – deferred income tax expense – deferred income tax expense) divided by the nominal income tax rate. The *LRATE*_*DIFF* is obtained by averaging the *RATE*_*DIFF* over five years (from year t-4 to year t) to provide a more comprehensive assessment of the difference between the nominal and effective income tax rates.

#### 4.2.3 Control variables.

In addition to population aging, there are other factors that can have an impact on corporate tax avoidance, and based on theoretical analysis and existing literature [51–52], this paper controls for two main types of variables: firm size (*SIZE*), Leverage (*LEV*), firm age (*AGE*), return on total assets (*ROA*), Tangible Assets (*TANG*), inventory (*INVENT*), Company Growth (*GROW*), Operating Cash Flow (*OCF*), shareholding of the first largest shareholder (*TOP1*), and Institutional Investors’ Shareholding Ratio (*INSHOLD*). Also, industry, year and province fixed effects are controlled for in the model. The specific variables are defined in Table 1.

**Table 1:** Definition of variables.

Variable Name	Variable Abbreviation	Variable Description
Population Aging	*OLD*	Ratio of persons aged 65 and over to total population, by region
	*OLD1*	Ratio of persons aged 65 and over to the working-age population aged 15–64, by region
Tax Avoidance	*BTD*	Accounting-tax differences, (total profit before tax – income tax expense/ nominal tax rate)/ total assets
	*DDBTD*	Accounting-tax differences on the effect of deduction of accrued profit
	*RATE_DIFF*	Difference between nominal income tax rate less effective income tax rate
	*LRATE_DIFF*	Five-year average of the difference between the nominal and effective income tax rates
Firm Size	*SIZE*	Total assets at end of period as natural logarithm
Leverage	*LEV*	Ratio of ending liabilities to total assets at the end of the period
Firm Age	*AGE*	Year of enterprise establishment to the current year plus 1 and take the logarithm
Return on Total Assets	*ROA*	Ratio of net income to total assets at the end of the period
Tangible Assets	*TANG*	Ratio of tangible assets to total assets at the end of the period
Inventory	*INVENT*	**The logarithm of (current year minus founding year plus one)**
Company Growth	*GROW*	Ratio of growth in operating income for the year to operating income at the beginning of the period
Operating Cash Flow	*OCF*	Cash flow from operating activities to total assets at the beginning of the period
Shareholding of the first Largest Shareholder	*TOP1*	The number of shares held by the shareholder with the largest shareholding in the year as a percentage of the total number of shares in the listed company
Institutional Investors’ Shareholding Ratio	*INSHOLD*	The number of shares held by institutional investors in the current year as a percentage of the total number of shares of listed companies
Industry	*INDUSTRY*	Industry dummy variables
Year	*YEAR*	Year dummy variable
Province	*PROVINCE*	Province dummy variables

### 4.3 Model construction

To test the effect of population aging on corporate tax avoidance, the following regression model is constructed:


$$TAXAVOID_{j,t}=\beta_{0}+\beta_{1}OLD_{p.t}+\sum {CONTROLS}_{i,t}+\sum {INDUSTRY+\sum YEAR+\sum PROVINCE+\varepsilon }_{i,t}$$ (1)

Among the variables considered in the analysis, the explanatory variable *TAXAVOID* serves as a proxy for corporate tax avoidance. The explanatory variable *OLD* represents population aging, while *CONTROLS* encompasses the control variables used in the regression analysis. Additionally, the inclusion of *INDUSTRY*, *YEAR*, and *PROVINCE* accounts for industry, year, and province fixed effects, respectively. This study specifically focuses on the coefficient β_1_ associated with population aging. A significantly negative β_1_ would indicate an inverse relationship between population aging and corporate tax avoidance, thereby supporting research hypothesis H1.

## 5. Empirical analysis

### 5.1 Descriptive statistics

Table 2 presents the descriptive statistics for the key variables employed in this study. Notably, population aging (*OLD*) exhibits significant heterogeneity across provinces, as evidenced by the range between the minimum (0.066) and maximum (0.196) values, representing a 2.97-fold disparity. Based on the UN’s population aging classification criteria, China as a whole entered the population aging stage in 2000, and all provinces covered in this analysis are essentially in the population aging stage throughout the sample period. The mean (0.120) and median (0.116) values of population aging demonstrate insignificant differences, suggesting a lack of pronounced right skewness. Regarding corporate tax avoidance, the mean (median) values for accounting-tax differences (*BTD*) and accounting-tax differences net of the effect of accrued profits (*DDBTD*) are 0.002 (0.000) and 0.002 (0.000), respectively, indicating minimal disparity between them. The descriptive statistics of the control variables align with existing literature.

**Table 2:** Descriptive statistics.

VARIABLE	N	MEAN	SD	MIN	P25	P50	P75	MAX
*OLD*	27,127	0.120	0.032	0.066	0.092	0.116	0.143	0.196
*BTD*	27,127	0.002	0.026	−0.068	−0.012	0.000	0.014	0.089
*DDBTD*	27,127	0.002	0.026	−0.073	−0.012	0.000	0.015	0.082
*SIZE*	27,127	22.397	1.304	20.065	21.465	22.210	23.142	26.438
*LEV*	27,127	0.427	0.194	0.061	0.274	0.424	0.574	0.852
*AGE*	27,127	2.929	0.335	1.099	2.708	2.944	3.178	4.190
*ROA*	27,127	0.051	0.040	0.002	0.022	0.041	0.070	0.203
*TANG*	27,127	0.215	0.159	0.002	0.091	0.183	0.305	0.696
*INVENT*	27,127	0.148	0.136	0.000	0.059	0.114	0.187	0.704
*GROW*	27,127	0.181	0.371	−0.459	−0.003	0.115	0.270	2.330
*OCF*	27,127	0.057	0.067	−0.135	0.017	0.054	0.095	0.252
*TOP1*	27,127	0.347	0.149	0.085	0.231	0.327	0.449	0.747
*INSHOLD*	27,127	0.460	0.242	0.004	0.276	0.482	0.651	0.913

### 5.2 Baseline regression analysis

Table 3 presents the baseline regression results for the association between population aging and corporate tax avoidance. Firm-level clustering robustness standard errors are employed to address the issue of heteroskedasticity. Columns (1) to (2) incorporate year, industry, and province fixed effects but exclude control variables, while columns (3) to (4) include both fixed effects and control variables. In columns (1) and (3), the explanatory variable is accounting-tax differences (*BTD*), while in columns (2) and (4), it is accounting-tax differences net of the effect of accrued profits (*DDBTD*). The findings reveal significant negative coefficients for population aging (*OLD*) in both column (1) (β_1_ = −0.055, t = −2.426) and column (3) (β_1_ = −0.054, t = −2.550) at the 5% level, even after controlling for the additional variables. Similarly, in column (2), the coefficient of population aging is significantly negative and significant at the 1% level (β_1_ = −0.069, T = −3.066), and in column (4), it remains significantly negative and significant at the 1% level (β_1_ = −0.063, T = −2.918) after introducing the control variables. These results indicate a significant negative relationship between the degree of population aging and corporate tax avoidance among listed companies. Specifically, population aging is found to reduce the extent of corporate tax avoidance, providing support for hypothesis H1 proposed in this study.

**Table 3:** Basic test results.

	(1)	(2)	(3)	(4)
VARIABLES	*BTD*	*DDBTD*	*BTD*	*DDBTD*
*OLD*	−0.055**	−0.069***	−0.054**	−0.063***
	(−2.426)	(−3.066)	(−2.550)	(−2.918)
*SIZE*			0.001*	0.000
			(1.769)	(1.295)
*LEV*			−0.006***	−0.006***
			(−3.329)	(−3.185)
*AGE*			0.005***	0.004***
			(5.064)	(4.134)
*ROA*			0.240***	0.164***
			(25.383)	(18.445)
*TANG*			0.012***	0.014***
			(5.359)	(6.340)
*INVENT*			−0.006**	−0.004
			(−2.560)	(−1.495)
*GROW*			−0.002***	−0.002***
			(−3.964)	(−3.857)
*OCF*			−0.039***	0.010***
			(−11.184)	(2.711)
*TOP1*			−0.006***	−0.007***
			(−2.613)	(−2.839)
*INSHOLD*			0.004***	0.005***
			(3.230)	(3.339)
*Constant*	0.030***	0.029***	−0.002	−0.000
	(6.211)	(5.557)	(−0.286)	(−0.026)
*Year FE*	YES	YES	YES	YES
*Industry FE*	YES	YES	YES	YES
*Province FE*	YES	YES	YES	YES
Observations	27127	27127	27,127	27,127
Adj.R^2^	0.078	0.075	0.193	0.152

Note: *, **, *** represent 10%, 5%, and 1% significance levels, respectively, with t-value tests for coefficients in parentheses. t-values are adjusted by firm-level CLUSTER and are kept consistent below.

Aligned with this study, Ferraro & Fiori [53] discovered that as the baby boomer generation in the United States ages, the impact of tax cuts on overall unemployment rates has been significantly reduced. Dynan [54] posits that population aging leads to a marked deterioration in fiscal conditions, necessitating an increase in tax revenue. Wang et al. [55] explored the influence of population aging on corporate digital transformation and found that the relationship is not linear but rather exhibits a U-shaped pattern. Distinguished from prior research, this paper quantitatively examines the mitigating effects and suppressive levels of population aging on corporate tax avoidance behavior from the corporate level.

### 5.3 Mechanism analysis

The baseline regression results suggest that population aging has a negative impact on corporate tax avoidance. However, further investigation is required to understand the underlying mechanisms driving this relationship. This study aims to explore the specific pathways through which population aging inhibits corporate tax avoidance, focusing on three dimensions: the factor substitution effect, innovation capability effect, and resource allocation effect.

#### 5.3.1 Factor substitution effect.

To examine the factor substitution effect, this study adopts a method employed by Zwick and Mahon [56] and examines the relationship between the level of population aging and indicators of capital-labor substitution. The variables of interest primarily include fixed asset investment (*INVEST*) and the count of non-R&D employees (*LABOR*). Fixed asset investment is calculated as (current fixed assets – previous fixed assets + annual depreciation)/ total assets, while the count of non-R&D employees is computed as (total employee count – R&D employee count)/ total employee count. Table 4 presents the results of the analysis examining the relationship between population aging and indicators of capital-labor substitution. In column (1), the coefficient between population aging and fixed asset investment (*INVEST*) is significantly positive at the 1% level, indicating that an increase in population aging significantly enhances firms’ investment in fixed assets. Specifically, a 1% increase in population aging leads to a corresponding increase in fixed asset investment by 10.010% of total assets. In column(2), the coefficient between population aging and non-R&D employee count (*LABOR*) is significantly negative at the 10% level, suggesting that an increase in population aging significantly reduces firms’ count of non-R&D employees. Specifically, a 1% increase in population aging leads to a decrease of 10.020% in non-R&D employee count. Through comprehensive analysis, the findings indicate that the rising level of population aging contributes to increased labor costs, lowers the relative price of capital, and prompts firms to increase their investment in fixed assets while substituting non-R&D employees. These results support the existence of the “factor substitution effect” channel, which serves as a significant pathway through which population aging inhibits tax avoidance by firms.

**Table 4:** Mechanism test results.

	Factor Substitution Effect		Innovation Capacity Effect			Resource Allocation Effect	
	(1)	(2)	(3)	(4)	(5)	(6)	(7)
VARIABLES	*INVEST*	*LABOR*	*RDSPEND*	*PATENT*	*PATP*	*TFP_LP*	*TFP_OP*
*OLD*	0.106***	−0.156*	4.447***	2.517***	1.904***	1.168***	1.865***
	(4.245)	(−1.795)	(2.671)	(3.258)	(3.098)	(3.227)	(6.115)
*SIZE*	0.002***	−0.009***	−0.060***	0.081***	−0.004	0.596***	0.425***
	(8.620)	(−3.200)	(−3.653)	(10.079)	(−0.617)	(61.712)	(52.204)
*LEV*	0.007***	−0.024**	−2.330***	0.038	−0.053	1.104***	0.881***
	(4.230)	(−2.382)	(−18.696)	(0.732)	(−1.329)	(18.028)	(16.792)
*AGE*	−0.011***	−0.062***	−0.921***	−0.050*	−0.064***	0.016	0.024
	(−12.395)	(−4.493)	(−15.788)	(−1.785)	(−3.113)	(0.544)	(0.982)
*ROA*	0.004	−0.005	−0.628	0.414*	0.355**	3.015***	2.606***
	(0.649)	(−0.162)	(−1.156)	(1.840)	(1.999)	(12.098)	(12.143)
*TANG*	0.018***	0.035***	−1.250***	0.057	−0.228***	−1.558***	−1.391***
	(9.178)	(3.004)	(−10.177)	(0.993)	(−5.182)	(−19.278)	(−20.463)
*INVENT*	−0.044***	0.038***	−1.511***	0.041	−0.036	1.034***	0.906***
	(−21.100)	(2.868)	(−10.714)	(0.639)	(−0.711)	(9.239)	(9.613)
*GROW*	0.007***	−0.003*	−0.027	−0.039**	−0.007	0.073***	0.112***
	(10.047)	(−1.789)	(−0.670)	(−2.170)	(−0.508)	(2.595)	(4.603)
*OCF*	0.015***	−0.005	0.593**	0.074	0.080	1.612***	1.065***
	(4.179)	(−0.443)	(2.260)	(0.640)	(0.835)	(8.944)	(6.835)
*TOP1*	−0.009***	−0.005	−0.459***	−0.066	−0.069	−0.083	−0.024
	(−5.228)	(−0.291)	(−3.652)	(−1.150)	(−1.510)	(−1.226)	(−0.419)
*INSHOLD*	0.001	0.053***	−0.551***	0.135***	0.069**	0.141***	0.062**
	(0.595)	(4.761)	(−5.999)	(3.312)	(2.042)	(3.845)	(1.989)
*Constant*	0.028***	0.296***	4.507***	−1.834***	0.078	−6.069***	−3.958***
	(4.273)	(4.214)	(10.172)	(−8.802)	(0.493)	(−27.642)	(−21.285)
*Year FE*	YES	YES	YES	YES	YES	YES	YES
*Industry FE*	YES	YES	YES	YES	YES	YES	YES
*Province FE*	YES	YES	YES	YES	YES	YES	YES
Observations	27,127	27,127	27,127	27,127	27,127	27,127	27,127
Adj.R^2^	0.381	0.415	0.501	0.102	0.077	0.395	0.351

#### 5.3.2 Innovation capability effect.

To examine the innovation capability effect, this study employs indicators such as research and development (R&D) expenditure (*RDSPEND*), patent applications (*PATENT*), and per capita patent applications (*PATP*). *RDSPEND* represents the ratio of R&D expenses to operating revenue, while *PATENT* measures the logarithm of the number of patent applications in a given year. *PATP* is defined as the number of patent applications divided by the number of employees in thousands. Table 4 presents the results of the analysis examining the relationship between population aging and firms’ research and development innovation. In column (3), the coefficient between population aging and R&D expenditure (*RDSPEND*) is significantly positive at the 1% level, indicating that an increase in population aging significantly enhances firms’ investment in R&D. In column (4), the coefficient between population aging and patent applications (*PATENT*) is significantly positive at the 1% level, suggesting that population aging significantly improves firms’ innovation output. In column (5), the coefficient between population aging and per capita patent applications (*PATP*) is significantly positive at the 1% level, indicating that population aging drives firms’ per capita innovation output. Considering these three indicators of R&D innovation, it becomes evident that population aging reduces the labor supply level and increases labor costs, which prompts firms to increase their investment in R&D and significantly enhance their innovation capabilities. Consequently, this mitigates the negative impact of declining labor supply and alleviates firms’ motives for tax avoidance. These findings confirm the existence of the “innovation capability effect” mechanism.

#### 5.3.3 Resource allocation effect.

This study utilizes the LP method and OP method to measure firms’ total factor productivity (*TFP*) and investigate the resource allocation effect. Table 4 presents the results of the analysis examining the relationship between population aging and firms’ TFP, measured by the LP (*TFP*_*LP*) and OP (*TFP*_*OP*) methods. In columns (6) and (7), the coefficients between population aging and TFP indicators (*TFP*_*LP* and *TFP*_*OP*) are both significantly positive at the 1% level. These findings indicate that population aging significantly enhances firms’ total factor productivity. The combined results suggest that population aging prompts firms to actively allocate capital in place of labor, leading to increased investment and output in research and development. As a result, firms’ total factor productivity improves, mitigating the negative effects of declining labor supply and reducing their motivation for tax avoidance. Hence, this study provides confirmation of the existence of the “resource allocation effect” mechanism.

### 5.4 Heterogeneity analysis

The impact of population aging on corporate tax avoidance may vary depending on the characteristics of listed companies, such as group affiliation and regional factors. To provide a comprehensive understanding of the relationship between population aging and tax avoidance, this paper conducts a detailed analysis considering various factors, including enterprise characteristics, labor costs, financing ability, financial status, enterprise taxation environment, and external factors.

#### 5.4.1 Types of business elements.

Capital-intensive firms have successfully substituted capital for labor, resulting in a lower demand for regular labor compared to labor-intensive firms. As a result, they experience a relatively smaller impact from population aging. Conversely, labor-intensive firms rely on a larger workforce, particularly employing a higher number of low-skilled workers. However, population aging reduces the size of the labor force, increases labor costs, and compels labor-intensive firms to invest in capital or innovation to mitigate labor costs, thus suppressing their motivation for tax avoidance. Therefore, this study anticipates that population aging will have a more pronounced inhibitory effect on tax avoidance in enterprises characterized by higher labor intensity or lower capital intensity.

To examine the impact of population aging on tax avoidance across different factor types, this study follows the approach of Zwick and Mahon [56]. Labor intensity (*LABOR*) is defined as the ratio of the number of employees to year-end fixed assets, while capital intensity is measured using the natural logarithm of year-end fixed assets (*CAP*). Interaction terms between these intensity measures and population aging are constructed and included in regression analyses. A value of 1 is assigned to the indicators of labor intensity and capital intensity when they exceed the industry annual median, and 0 otherwise. Table 5 presents the regression results incorporating labor intensity and capital intensity. In columns (1) and (2), the interaction term between labor intensity measure and population aging (LABOR*OLD) is significantly positive, with significance observed at the 5% level. This indicates that the inhibitory effect of population aging on tax avoidance is more pronounced in firms with higher labor intensity. In columns (3) and (4), the interaction term between capital intensity and population aging (CAP*OLD) is significantly positive, with significance observed at least at the 10% level. This suggests that population aging has a more significant inhibitory effect on tax avoidance in firms with lower capital intensity.

**Table 5:** Types of business elements.

	Labor-Intensive		Capital-Intensive	
	(1)	(2)	(3)	(4)
VARIABLES	*BTD*	*DDBTD*	*BTD*	*DDBTD*
*OLD*	−0.039**	−0.046**	−0.064***	−0.074***
	(−2.144)	(−2.473)	(−3.493)	(−3.969)
LABOR*OLD	−0.024**	−0.029***		
	(−2.369)	(−2.720)		
*LABOR*	0.004***	0.005***		
	(3.115)	(3.606)		
CAP*OLD			0.017*	0.019*
			(1.711)	(1.842)
*CAP*			−0.003**	−0.003**
			(−2.366)	(−2.490)
*SIZE*	0.001***	0.000***	0.001***	0.001***
	(3.644)	(2.884)	(3.952)	(3.213)
*LEV*	−0.006***	−0.005***	−0.006***	−0.006***
	(−5.095)	(−4.691)	(−5.277)	(−4.930)
*AGE*	0.005***	0.004***	0.005***	0.004***
	(9.404)	(7.837)	(9.408)	(7.816)
*ROA*	0.241***	0.164***	0.240***	0.163***
	(37.786)	(26.471)	(37.741)	(26.368)
*TANG*	0.011***	0.014***	0.013***	0.016***
	(9.205)	(10.982)	(9.643)	(11.320)
*INVENT*	−0.006***	−0.003**	−0.006***	−0.004**
	(−4.072)	(−2.243)	(−4.249)	(−2.439)
*GROW*	−0.002***	−0.002***	−0.002***	−0.002***
	(−4.053)	(−3.954)	(−4.151)	(−4.076)
*OCF*	−0.039***	0.010***	−0.039***	0.010***
	(−12.970)	(3.119)	(−12.967)	(3.127)
*TOP1*	−0.006***	−0.006***	−0.006***	−0.007***
	(−4.629)	(−5.194)	(−4.684)	(−5.256)
*INSHOLD*	0.004***	0.005***	0.004***	0.005***
	(5.307)	(5.776)	(5.365)	(5.833)
*Constant*	−0.007	−0.006	−0.007	−0.005
	(−1.456)	(−1.196)	(−1.324)	(−0.911)
*Year FE*	YES	YES	YES	YES
*Industry FE*	YES	YES	YES	YES
*Province FE*	YES	YES	YES	YES
Observations	27,127	27,127	27,127	27,127
Adj.R^2^	0.192	0.153	0.192	0.152

#### 5.4.2 Financing capacity.

1. Financing constraints

The practice of tax avoidance exhibits a “cash flow effect”, whereby companies can reduce their tax payments through such activities, thereby increasing their free cash flow and alleviating financing constraints [57]. Companies facing higher financing constraints encounter greater difficulties and higher costs in securing external financing. They also exhibit a higher demand for cash flow and a greater likelihood of facing financial risks, resulting in a stronger motivation for tax avoidance. Population aging contributes to elevated labor costs. According to the theory of factor substitution, companies tend to substitute labor with capital and increase their investment in research and development (R&D) innovation to enhance overall management efficiency, improve product competitiveness, and increase profitability. Consequently, this helps alleviate financing constraints and reduces the motivation for tax avoidance. Thus, this study expects that population aging will exert a more pronounced inhibitory effect on tax avoidance in firms characterized by higher financing constraints.

To examine the impact of population aging on tax avoidance under varying levels of financing constraints, this study used the KZ index and the WW index as measures of firms’ financing constraints, interaction terms between these indices and population aging are constructed for regression analysis. A higher KZ/WW index denotes a greater degree of financing constraints, with a value of 1 assigned when the constraints exceed the industry’s annual median, and 0 otherwise. Table 6 presents the regression results accounting for the level of financing constraints. In columns (1) and (2), the interaction term between the financing constraint indicator and population aging (KZ*OLD) is significantly negative at the 1% level, implying a more pronounced inhibitory effect of population aging on tax avoidance in highly financially constrained listed firms. Similarly, in columns (3) and (4), the interaction term between the financing constraint indicator and population aging (WW*OLD) is significantly negative at the 5% level, indicating a more substantial inhibitory effect of population aging on tax avoidance in highly financially constrained listed firms.

**Table 6:** Financing constraints and financing costs.

	KZ Index		WW Index		Financing Costs	
	(1)	(2)	(3)	(4)	(5)	(6)
VARIABLES	*BTD*	*DDBTD*	*BTD*	*DDBTD*	*BTD*	*DDBTD*
*OLD*	−0.002	−0.021	−0.029	−0.043**	−0.024	−0.034*
	(−0.114)	(−1.007)	(−1.512)	(−2.248)	(−1.334)	(−1.805)
KZ*OLD	−0.061***	−0.050***				
	(−4.910)	(−3.957)				
*KZ*	0.008***	0.007***				
	(5.621)	(4.552)				
WW*OLD			−0.031***	−0.025**		
			(−2.787)	(−2.135)		
*WW*			0.004***	0.003**		
			(3.294)	(2.513)		
FINCOST*OLD					−0.051***	−0.050***
					(−4.945)	(−4.815)
*FINCOST*					0.007***	0.006***
					(5.502)	(5.013)
*SIZE*	0.001***	0.000***	0.001***	0.001***	0.001***	0.000***
	(3.491)	(2.589)	(3.863)	(2.860)	(3.490)	(2.582)
*LEV*	−0.007***	−0.007***	−0.006***	−0.006***	−0.007***	−0.006***
	(−6.033)	(−5.575)	(−5.341)	(−5.003)	(−5.820)	(−5.193)
*AGE*	0.005***	0.004***	0.005***	0.004***	0.005***	0.004***
	(9.108)	(7.541)	(9.281)	(7.683)	(9.123)	(7.543)
*ROA*	0.240***	0.163***	0.240***	0.163***	0.240***	0.163***
	(37.720)	(26.352)	(37.756)	(26.380)	(37.719)	(26.311)
*TANG*	0.011***	0.014***	0.012***	0.014***	0.011***	0.014***
	(9.180)	(11.083)	(9.605)	(11.458)	(9.005)	(11.024)
*INVENT*	−0.007***	−0.004***	−0.006***	−0.004**	−0.006***	−0.004**
	(−4.461)	(−2.606)	(−4.085)	(−2.299)	(−4.219)	(−2.428)
*GROW*	−0.002***	−0.002***	−0.002***	−0.002***	−0.002***	−0.002***
	(−4.112)	(−4.038)	(−3.873)	(−3.853)	(−4.193)	(−4.097)
*OCF*	−0.037***	0.012***	−0.039***	0.010***	−0.039***	0.010***
	(−11.895)	(3.656)	(−12.845)	(3.205)	(−12.893)	(3.177)
*TOP1*	−0.006***	−0.006***	−0.006***	−0.007***	−0.006***	−0.006***
	(−4.622)	(−5.204)	(−4.706)	(−5.278)	(−4.543)	(−5.180)
*INSHOLD*	0.004***	0.005***	0.004***	0.005***	0.004***	0.005***
	(5.272)	(5.747)	(5.362)	(5.822)	(5.284)	(5.744)
*Constant*	−0.009**	−0.006	−0.009*	−0.006	−0.007	−0.005
	(−2.000)	(−1.281)	(−1.869)	(−1.134)	(−1.513)	(−0.985)
*Year FE*	YES	YES	YES	YES	YES	YES
*Industry FE*	YES	YES	YES	YES	YES	YES
*Province FE*	YES	YES	YES	YES	YES	YES
Observations	27,127	27,127	27,127	27,127	27,127	27,127
Adj.R^2^	0.193	0.153	0.192	0.152	0.193	0.153

2. Financing costs

Aligned with the rationale of financing constraints, firms encountering higher financing costs possess a greater incentive to engage in tax avoidance practices, enabling them to preserve resources for future investments. However, population aging diminishes firms’ motivations for tax avoidance, and this impact is anticipated to be more prominent in firms grappling with higher financing costs. Accordingly, this study postulates that population aging exerts a more significant inhibitory effect on tax avoidance among firms burdened with higher financing costs.

To explore the influence of population aging on tax avoidance across various financing cost levels, this study defines financing costs (*FINCOST*) as the ratio of financial expenses to total liabilities for listed companies. A higher value of *FINCOST* indicates elevated financing costs. The variable is assigned a value of 1 when financing costs exceed the industry’s annual median and 0 otherwise. Building upon the baseline regression model (1), we investigate the interaction effects using the FINCOST*OLD term. Table 6 presents the regression results incorporating the financing cost indicator. In columns (5) to (6), the interaction term between financing costs and population aging (FINCOST*OLD) is significantly negative at the 1% level, implying a more pronounced inhibitory effect of population aging on tax avoidance in firms burdened with higher financing costs. These findings substantiate the hypothesis that population aging exerts a stronger influence on tax avoidance in firms facing elevated financing costs.

#### 5.4.3 Financial status.

1. Financial risk

Firms characterized by higher financial risks are more susceptible to financial distress and, therefore, exhibit a stronger inclination to engage in tax avoidance activities. By reducing their cash tax payments, these firms seek to fortify their financial position and alleviate the risks associated with financial distress, allowing them to allocate resources towards future investments. The inhibitory effect of population aging on firms’ motivation for tax avoidance is expected to be more pronounced in those facing higher financial risks, as they already possess a heightened initial motivation for tax avoidance. Consequently, this study proposes the hypothesis that population aging exerts a more significant inhibitory effect on tax avoidance in firms with higher financial risks.

To assess the impact of population aging on tax avoidance under varying levels of financial risk, this study employs the *ZSCORE* index and the *OSCORE* index as measures of financial risk. A lower value of the *ZSCORE* index signifies higher financial risk, while a higher value of the *OSCORE* index indicates increased financial risk. These variables are assigned a value of 1 when they surpass the industry median and 0 otherwise. Regression analysis is conducted using interaction terms between these indices and population aging. Table 7 presents the regression results incorporating the financial risk factors. In columns (1) and (2), the interaction term between financial risk and population aging (ZSCORE*OLD) is significantly positive at the 5% level, indicating a more pronounced inhibitory effect of population aging on tax avoidance in firms with higher financial risks. Similarly, in columns (3) and (4), the interaction term between financial risk and population aging (OSCORE*OLD) is significantly negative at the 1% level, demonstrating a more substantial inhibitory effect of population aging on tax avoidance in firms with higher financial risks.

**Table 7:** Corporate financial risk and operating cash flow.

	ZSCORE Index		OSCORE Index		Operating Cash Flow	
	(1)	(2)	(3)	(4)	(5)	(6)
VARIABLES	*BTD*	*DDBTD*	*BTD*	*DDBTD*	*BTD*	*DDBTD*
*OLD*	−0.073***	−0.082***	−0.023	−0.041**	−0.077***	−0.077***
	(−3.971)	(−4.369)	(−1.187)	(−2.073)	(−4.283)	(−4.216)
ZSCORE*OLD	0.026**	0.026**				
	(2.446)	(2.426)				
*ZSCORE*	−0.004***	−0.003**				
	(−2.883)	(−2.492)				
OSCORE*OLD			−0.042***	−0.030***		
			(−3.652)	(−2.593)		
*OSCORE*			0.004***	0.003**		
			(2.857)	(1.994)		
OCF*OLD					0.039***	0.023**
					(3.913)	(2.295)
*OCF*					−0.005***	−0.002
					(−3.688)	(−1.641)
*SIZE*	0.000***	0.000**	0.000***	0.000**	0.001***	0.000**
	(2.987)	(2.308)	(2.806)	(2.060)	(3.231)	(2.367)
*LEV*	−0.007***	−0.006***	−0.005***	−0.005***	−0.006***	−0.006***
	(−5.686)	(−5.013)	(−3.824)	(−3.766)	(−5.379)	(−5.024)
*AGE*	0.005***	0.004***	0.005***	0.004***	0.005***	0.004***
	(9.215)	(7.627)	(9.206)	(7.629)	(9.320)	(7.711)
*ROA*	0.241***	0.163***	0.239***	0.162***	0.239***	0.162***
	(37.561)	(26.146)	(37.432)	(26.107)	(37.648)	(26.274)
*TANG*	0.012***	0.014***	0.012***	0.014***	0.012***	0.014***
	(9.565)	(11.449)	(9.689)	(11.518)	(9.676)	(11.429)
*INVENT*	−0.006***	−0.004**	−0.006***	−0.004**	−0.006***	−0.004**
	(−4.182)	(−2.404)	(−4.194)	(−2.379)	(−4.208)	(−2.396)
*GROW*	−0.002***	−0.002***	−0.002***	−0.002***	−0.002***	−0.002***
	(−4.183)	(−4.090)	(−4.184)	(−4.096)	(−4.154)	(−4.050)
*OCF*	−0.039***	0.010***	−0.040***	0.009***	–	–
	(−12.902)	(3.164)	(−13.102)	(2.954)	–	–
*TOP1*	−0.006***	−0.007***	−0.006***	−0.007***	−0.006***	−0.007***
	(−4.764)	(−5.303)	(−4.662)	(−5.247)	(−4.743)	(−5.281)
*INSHOLD*	0.005***	0.005***	0.004***	0.005***	0.004***	0.005***
	(5.427)	(5.811)	(5.345)	(5.809)	(5.335)	(5.759)
*Constant*	0.001	0.002	−0.004	−0.002	0.000	0.001
	(0.301)	(0.499)	(−0.937)	(−0.363)	(0.084)	(0.220)
*Year FE*	YES	YES	YES	YES	YES	YES
*Industry FE*	YES	YES	YES	YES	YES	YES
*Province FE*	YES	YES	YES	YES	YES	YES
Observations	27,127	27,127	27,127	27,127	27,127	27,127
Adj.R^2^	0.192	0.152	0.192	0.152	0.192	0.152

2. Operating cash flow

Firms characterized by lower levels of operating cash flow are more susceptible to financial risks and are at a greater risk of experiencing financial distress. These firms exhibit a stronger motivation to minimize cash tax payments, thereby demonstrating a heightened inclination for tax avoidance. Given the already elevated initial motivation for tax avoidance in firms with lower operating cash flow, the inhibitory effect of population aging on their tax avoidance motivation is anticipated to be more pronounced. Consequently, this study hypothesizes that population aging exerts a more significant inhibitory effect on tax avoidance in firms with lower operating cash flow.

To investigate the impact of population aging on tax avoidance across different levels of operating cash flow, this study constructs an operating cash flow indicator (*OCF*) and conducts regression analysis to examine its interaction with population aging. A higher value of the *OCF* indicates a greater level of operating cash flow. The variable is assigned a value of 1 when the operating cash flow surpasses the industry’s annual median, and 0 otherwise. The regression results considering the operating cash flow indicator are presented in Table 7. In columns (5) and (6), the interaction term between operating cash flow and population aging (OCF*OLD) is significantly positive at the 5% level, indicating a more pronounced inhibitory effect of population aging on tax avoidance in firms with lower levels of operating cash flow. These findings suggest that population aging holds a stronger influence on tax avoidance in firms characterized by lower operating cash flow levels.

#### 5.4.4 External financial environment.

In regions characterized by a favorable external financing environment, financial institutions possess greater capacity and incentive to discern and supervise corporate financing activities. This, in turn, alleviates the constraints on financing channels and distorting funding costs caused by asymmetric information in credit markets. The enhanced efficiency of capital allocation and the mitigation of corporate financing constraints result in reduced incentives for tax avoidance among businesses. Therefore, this study postulates that population aging will have a more pronounced inhibitory effect on the extent of tax avoidance in enterprises operating in regions with a less favorable external financing environment.

To examine the impact of population aging on corporate tax avoidance under different external financing environments, we draw inspiration from the research by Chen et al. [58] and measure the external financing environment (*FINANCE*) using the financial marketization index within the Marketization Index Report for Chinese Provinces (2021). Following the baseline regression model (1), we employ an approach involving interaction terms for empirical analysis. A higher financial marketization index indicates a more favorable external financing environment, with a value of 1 assigned when the index exceeds the industry median, and 0 otherwise. The empirical results for the external financing environment are presented in Table 8. In columns (3) and (4), the interaction term between the external financing environment and population aging (FINANCE*OLD) exhibits significant positive coefficients at the 5% significance level, indicating a more pronounced inhibitory effect of population aging on corporate tax avoidance in listed companies operating in regions with a less favorable external financing environment.

**Table 8:** External financial environment.

	(1)	(2)
VARIABLES	*BTD*	*DDBTD*
*OLD*	−0.065***	−0.072***
	(−3.287)	(−3.589)
FINANCE*OLD	0.040**	0.041**
	(2.071)	(2.121)
*FINANCE*	−0.004	−0.003
	(−1.452)	(−1.320)
*SIZE*	0.001***	0.000**
	(3.223)	(2.361)
*LEV*	−0.006***	−0.006***
	(−5.358)	(−5.019)
*AGE*	0.005***	0.004***
	(9.140)	(7.532)
*ROA*	0.240***	0.163***
	(37.662)	(26.284)
*TANG*	0.012***	0.014***
	(9.562)	(11.410)
*INVENT*	−0.006***	−0.004**
	(−4.161)	(−2.344)
*GROW*	−0.002***	−0.002***
	(−4.156)	(−4.082)
*OCF*	−0.039***	0.010***
	(−12.942)	(3.144)
*TOP1*	−0.006***	−0.007***
	(−4.715)	(−5.288)
*INSHOLD*	0.004***	0.005***
	(5.388)	(5.870)
*Constant*	−0.001	0.001
	(−0.151)	(0.150)
*Year FE*	YES	YES
*Industry FE*	YES	YES
*Province FE*	YES	YES
Observations	27,127	27,127
Adj.R^2^	0.192	0.152

### 5.5 Robustness test

To ensure the robustness and reliability of the empirical findings in this study, various methods have been employed to conduct rigorous robustness checks and address potential endogeneity concerns in the baseline model. These methods encompass conducting persistence tests, altering the measurement of explanatory and dependent variables, excluding outliers, performing balanced panel data regressions, and addressing endogeneity issues.

#### 5.5.1 Continuity test.

Considering the possibility of lagged effects of population aging on firm behavior at the micro-level, where the impact of population aging may manifest in the tax avoidance indicators of subsequent periods, this study addresses this concern by introducing a one-period lag of population aging in the regression analysis. This lagged approach helps alleviate concerns regarding reverse causality [59]. The regression results incorporating the lagged one-period population aging variable are presented in Table 9. In columns (1) and (2), the lagged population aging variable remains significant and negative, with significance levels of at least 10%. This finding suggests the presence of a persistent inhibitory effect of population aging on corporate tax avoidance, further affirming the robustness of the conclusions derived from the baseline regression analysis.

**Table 9:** Persistence test and replacement variable metric.

	Continuity Test		Changing the Dependent Variable Measure		Changing the Independent Variable Measure	
	(1)	(2)	(3)	(4)	(5)	(6)
VARIABLES	*BTD*_*t + 1*_	*DDBTD*_*t + 1*_	*RATE_DIFF*	*LRATE_DIFF*	*BTD*	*DDBTD*
*OLD*	−0.041*	−0.063***	−0.199**	−0.130*		
	(−1.769)	(−2.675)	(−2.254)	(−1.869)		
*OLD1*					−0.028**	−0.034**
					(−2.076)	(−2.474)
*SIZE*	0.001**	0.001	0.004***	0.006***	0.001*	0.000
	(2.173)	(1.585)	(3.937)	(6.232)	(1.783)	(1.310)
*LEV*	−0.008***	−0.007***	−0.039***	−0.048***	−0.006***	−0.006***
	(−4.036)	(−3.687)	(−4.946)	(−6.563)	(−3.353)	(−3.226)
*AGE*	0.005***	0.004***	0.006	−0.000	0.005***	0.004***
	(4.537)	(3.568)	(1.550)	(−0.076)	(5.069)	(4.139)
*ROA*	0.243***	0.166***	0.802***	0.422***	0.240***	0.163***
	(22.921)	(16.792)	(26.616)	(18.559)	(25.271)	(18.407)
*TANG*	0.011***	0.014***	0.015*	0.004	0.012***	0.014***
	(4.459)	(5.469)	(1.662)	(0.469)	(5.366)	(6.351)
*INVENT*	−0.006**	−0.004	−0.031***	−0.036***	−0.006**	−0.004
	(−2.398)	(−1.477)	(−2.771)	(−3.828)	(−2.544)	(−1.476)
*GROW*	−0.002***	−0.002***	0.005**	0.001	−0.002***	−0.002***
	(−4.007)	(−4.129)	(2.444)	(0.782)	(−3.879)	(−3.833)
*OCF*	−0.045***	0.004	−0.144***	−0.099***	−0.039***	0.010***
	(−11.619)	(1.014)	(−10.481)	(−9.596)	(−11.164)	(2.722)
*TOP1*	−0.006**	−0.008***	−0.002	0.007	−0.006***	−0.007***
	(−2.573)	(−3.162)	(−0.230)	(0.982)	(−2.591)	(−2.822)
*INSHOLD*	0.005***	0.006***	0.014***	0.005	0.004***	0.005***
	(3.252)	(3.654)	(2.798)	(1.234)	(3.199)	(3.336)
*Constant*	−0.007	−0.003	−0.003	−0.007	−0.005	−0.003
	(−0.847)	(−0.295)	(−0.090)	(−0.260)	(−0.617)	(−0.363)
*Year FE*	YES	YES	YES	YES	YES	YES
*Industry FE*	YES	YES	YES	YES	YES	YES
*Province FE*	YES	YES	YES	YES	YES	YES
Observations	21,613	21,613	27,127	27,127	27,127	27,127
Adj.R^2^	0.199	0.156	0.148	0.158	0.192	0.152

**Table 10:** Using subsamples.

	Remove Samples from the Liquor and Real Estate Industries		Remove the Sample of Central Enterprises		Remove Large City Sample	
	(1)	(2)	(3)	(4)	(5)	(6)
VARIABLES	BTD	DDBTD	BTD	DDBTD	BTD	BTD
*OLD*	−0.058***	−0.063***	−0.054**	−0.063***	−0.060***	−0.071***
	(−2.579)	(−2.788)	(−2.468)	(−2.867)	(−2.669)	(−3.121)
*SIZE*	0.001*	0.000	0.000	0.000	0.000	0.000
	(1.920)	(1.321)	(1.635)	(1.122)	(0.986)	(0.241)
*LEV*	−0.005***	−0.005***	−0.006***	−0.006***	−0.005***	−0.005***
	(−2.737)	(−2.644)	(−3.145)	(−3.068)	(−2.893)	(−2.586)
*AGE*	0.005***	0.005***	0.005***	0.004***	0.006***	0.005***
	(5.529)	(4.653)	(5.059)	(4.102)	(5.902)	(5.173)
*ROA*	0.250***	0.172***	0.240***	0.162***	0.243***	0.166***
	(25.737)	(19.147)	(25.114)	(18.265)	(24.486)	(18.031)
*TANG*	0.011***	0.014***	0.012***	0.014***	0.011***	0.013***
	(5.123)	(5.963)	(5.259)	(6.202)	(4.671)	(5.768)
*INVENT*	−0.008***	−0.004	−0.007***	−0.004	−0.007***	−0.004*
	(−2.951)	(−1.621)	(−2.678)	(−1.582)	(−2.782)	(−1.667)
*GROW*	−0.002***	−0.002***	−0.002***	−0.002***	−0.002***	−0.002***
	(−3.528)	(−3.253)	(−3.731)	(−3.636)	(−3.903)	(−3.921)
*OCF*	−0.039***	0.012***	−0.039***	0.010***	−0.039***	0.010***
	(−10.097)	(3.147)	(−11.025)	(2.676)	(−10.588)	(2.680)
*TOP1*	−0.005**	−0.005**	−0.006***	−0.007***	−0.007***	−0.008***
	(−2.075)	(−2.198)	(−2.649)	(−2.854)	(−3.031)	(−3.216)
*INSHOLD*	0.004***	0.005***	0.004***	0.005***	0.005***	0.006***
	(3.188)	(3.170)	(3.057)	(3.248)	(3.599)	(3.846)
*Constant*	−0.004	−0.002	−0.001	0.001	0.001	0.004
	(−0.570)	(−0.297)	(−0.183)	(0.135)	(0.104)	(0.491)
*Year FE*	YES	YES	YES	YES	YES	YES
*Industry FE*	YES	YES	YES	YES	YES	YES
*Province FE*	YES	YES	YES	YES	YES	YES
Observations	25,436	25,436	26,591	26,591	24,837	24,837
Adj.R^2^	0.193	0.151	0.188	0.148	0.194	0.154

#### 5.5.2 Changing the dependent variable measure.

To ensure the robustness of the baseline results, this study considers the potential influence of different measurements of the dependent variable on corporate tax avoidance. Alternative measures, namely the difference between the nominal tax rate and the effective tax rate (*RATE*_*DIFF*) and the five-year average of this difference (*LRATE*_*DIFF*), are incorporated. Please refer to Table 1 for specific variable definitions. The regression results incorporating these alternative measures of corporate tax avoidance are presented in Table 9. In columns (3) and (4), the coefficient of population aging remains significantly negative, with a significance level of 10%. This implies that the negative impact of population aging on corporate tax avoidance remains statistically significant. These findings provide support for the hypotheses posited in this study.

#### 5.5.3 Changing the independent variable measure.

Additionally, as an alternative measure reflecting population aging, the old-age dependency ratio is considered. This study utilizes the old-age dependency ratio (*OLD1*) as an explanatory variable to further examine the impact of population aging on corporate tax avoidance. The regression results incorporating this variable are presented in Table 9. In columns (5) to (6), the coefficient of the old-age dependency ratio (*OLD1*) is significantly negative, with a significance level of 5%. This suggests that the negative impact of population aging on corporate tax avoidance remains statistically significant. These findings affirm the robustness of the conclusions drawn from the baseline regression analysis. By incorporating the old-age dependency ratio as an alternative measure of population aging, this study provides additional evidence of the significant negative effect of population aging on corporate tax avoidance, further reinforcing the robustness of the findings derived from the baseline regression model.

#### 5.5.4 Using subsamples.

1. Industry sample processing

To mitigate potential interference from specific tax types and tax policies on the data sample, this study employs two different sample treatments in the regression analysis. Firstly, the sample is restricted by excluding industries subject to higher consumption taxes, such as the liquor industry, and industries subject to higher property value-added taxes, such as the real estate industry. Secondly, a separate regression analysis is conducted using a sample comprising only the manufacturing sector. The regression results after implementing these industry sample treatments are presented in column (1) and (2) in Table 10. The findings indicate that in both sample treatments, the coefficient of population aging remains significantly negative, with a significance level of at least 1%. This suggests that the negative impact of population aging on corporate tax avoidance remains statistically significant, thereby reinforcing the reliability of the results. By conducting regressions with different industry sample treatments, this study addresses concerns related to specific tax regimes and tax policies, and affirms the significant negative effect of population aging on corporate tax avoidance. The robustness of the results is confirmed.

2. City and enterprise sample processing

Given the substantial regional disparities in economic development in China and the potential influence of population mobility in metropolitan areas, the impact of population aging on large cities may be relatively smaller. Furthermore, state-owned enterprises (SOEs), such as central enterprises, carry greater social responsibilities compared to other types of enterprises and may adopt different approaches to address population aging issues. Therefore, this study employs sample treatments that exclude observations from major cities such as Beijing, Shanghai, Guangzhou, and Shenzhen, as well as observations from SOEs, in the regression analyses. The regression results after implementing these sample treatments based on city and ownership structure are reported in Table 10. The findings indicate that in columns (3) to (6), after removing observations from SOEs and major cities, the coefficient of population aging remains significantly negative, with a significance level of at least 5%. This implies that the negative impact of population aging on corporate tax avoidance remains statistically significant, confirming the robustness of the results. By conducting regressions with different sample treatments based on cities and ownership structure, this study addresses concerns related to population mobility and ownership structure, and verifies the significant negative effect of population aging on corporate tax avoidance. The robustness of the results is upheld.

#### 5.5.5 Endogeneity test.

1. Corporate and high-dimensional fixed effects

To account for firm-specific characteristics and address potential concerns regarding omitted variables, this study incorporates controls for “year × industry” interaction fixed effects, and “industry × province” interaction fixed effects. By including these high-order joint fixed effects, the regression model takes into consideration various unobserved factors that may influence the examined relationship. The regression results after controlling for “time × industry” interaction fixed effects, and “industry × province” interaction fixed effects are presented in Table 11. The findings indicate that even after incorporating these additional controls, the coefficient of population aging remains significantly negative, with a significance level of 10%. This implies that the negative impact of population aging on corporate tax avoidance remains statistically significant, and the conclusions drawn from the baseline regression remain unchanged. By accounting for firm-specific characteristics and employing high-order joint fixed effects, this study further enhances the validity and robustness of the findings regarding the negative effect of population aging on corporate tax avoidance.

**Table 11:** Fixed effects.

	“Year × Industry” Fixed Effect		“Industry × Province” Fixed Effect	
	(1)	(2)	(3)	(4)
VARIABLES	*BTD*	*DDBTD*	*BTD*	*DDBTD*
*OLD*	−0.040*	−0.046**	−0.051**	−0.057***
	(−1.853)	(−2.092)	(−2.450)	(−2.714)
*SIZE*	0.001**	0.000	0.001**	0.001*
	(2.046)	(1.530)	(2.061)	(1.742)
*LEV*	−0.007***	−0.006***	−0.005***	−0.006***
	(−3.707)	(−3.528)	(−2.894)	(−2.992)
*AGE*	0.005***	0.004***	0.005***	0.005***
	(4.840)	(3.924)	(5.558)	(4.401)
*ROA*	0.236***	0.159***	0.251***	0.171***
	(24.314)	(17.416)	(26.859)	(19.685)
*TANG*	0.011***	0.014***	0.010***	0.013***
	(5.042)	(6.060)	(4.539)	(5.509)
*INVENT*	−0.008***	−0.005**	−0.006**	−0.002
	(−3.118)	(−1.998)	(−2.151)	(−0.704)
*GROW*	−0.002***	−0.002***	−0.002***	−0.002***
	(−3.687)	(−3.787)	(−4.938)	(−4.635)
*OCF*	−0.039***	0.010***	−0.035***	0.013***
	(−10.903)	(2.658)	(−10.515)	(3.828)
*TOP1*	−0.005**	−0.006**	−0.005**	−0.005**
	(−2.186)	(−2.464)	(−2.378)	(−2.315)
*INSHOLD*	0.004***	0.005***	0.003**	0.004**
	(3.169)	(3.339)	(2.363)	(2.443)
*Constant*	−0.030***	−0.024***	−0.032***	−0.027***
	(−4.121)	(−3.185)	(−4.279)	(−3.464)
*Year FE*	YES	YES	YES	YES
*Industry FE*	YES	YES	YES	YES
*Province FE*	YES	YES	YES	YES
Year*Industry FE	YES	YES	NO	NO
Industry*Province FE	NO	NO	YES	YES
Observations	27,127	27,127	27,127	27,127
Adj.R^2^	0.197	0.155	0.244	0.207

2. Instrumental variables

Population aging, as a macro-level variable at the provincial level, does not pose issues of reverse causality between population aging and tax avoidance variables at the firm level. However, there may still be endogeneity concerns arising from omitted variables or measurement errors. To address these concerns, instrumental variable (IV) methods are employed in this study. Drawing on Acemoglu and Restrepo [44], the birth rate (*BIRTH*) for each region from 1965 to 1980 is used as an instrumental variable. The validity of an instrumental variable is usually subject to the conditions of relevance and exogeneity. First, the birth rates in these years determine the demographic structure of the population in the coming period, especially the share of the aging population, which satisfies the relevance requirement. Second, in terms of exogeneity, birth rates from decades ago are not directly linked to the existing random term. The regression results using the instrumental variable approach are reported in Table 12. In column (1), the coefficient of the instrument variable (*BIRTH*) is significantly negative and statistically significant at the 1% level, indicating a strong correlation between the instrumental variable and the explanatory variable. In columns (2) and (3), the coefficients of population aging are both significantly negative and statistically significant, suggesting that population aging continues to have a negative impact on tax avoidance by firms. These results effectively address endogeneity concerns and support the conclusions of this study.

**Table 12:** Instrumental variable method.

	Stage One	Stage Two	
	(1)	(2)	(3)
VARIABLES	*OLD*	*BTD*	*DDBTD*
*BIRTH*	0.000***		
	(4.034)		
*OLD*		−1.087*	−1.050**
		(−1.829)	(−2.256)
*SIZE*	−0.000	0.000***	0.000**
	(−0.570)	(2.768)	(2.078)
*LEV*	−0.001**	−0.007***	−0.007***
	(−2.283)	(−5.339)	(−5.441)
*AGE*	−0.001***	0.004***	0.003***
	(−3.872)	(4.922)	(4.719)
*ROA*	−0.001	0.239***	0.163***
	(−0.418)	(47.199)	(31.874)
*TANG*	−0.001	0.011***	0.014***
	(−1.588)	(7.909)	(10.173)
*INVENT*	0.001*	−0.005***	−0.003
	(1.921)	(−2.871)	(−1.550)
*GROW*	0.000	−0.002***	−0.002***
	(1.620)	(−4.216)	(−3.861)
*OCF*	−0.002	−0.040***	0.008***
	(−1.641)	(−13.473)	(2.616)
*TOP1*	−0.001***	−0.007***	−0.008***
	(−3.279)	(−4.306)	(−5.149)
*INSHOLD*	0.001**	0.005***	0.005***
	(2.132)	(5.198)	(5.806)
*Constant*	0.125***	0.133	0.128**
	(83.321)	(1.467)	(2.152)
*Year FE*	YES	YES	YES
*Industry FE*	YES	YES	YES
*Province FE*	YES	YES	YES
Observations	27,127	27,127	27,127
Adj.R^2^	0.915	0.072	0.043

## 6. Research conclusions and policy recommendations

### 6.1. Research conclusions

Tax avoidance is a prevalent economic activity among firms, which not only affects individual businesses but also leads to fiscal losses for the government at a national level. The increasing phenomenon of population aging has profound and wide-ranging impacts. Therefore, understanding the influence of population aging on tax avoidance by firms is of great practical importance. This study focuses on non-financial listed companies in China’s A-share market from 2008 to 2023, empirically examines the impact of population aging on tax avoidance by firms, and investigates the underlying mechanisms and heterogeneity effects. The research findings are as follows: (1) There is a significant negative correlation between population aging and tax avoidance by firms, indicating that as population aging increases, firms engage in less tax avoidance. This suggests that population aging significantly reduces firms’ motivation for tax avoidance. (2) The analysis of underlying mechanisms reveals that population aging increases labor costs for firms, leading them to substitute labor with capital, enhance innovation capabilities, and improve overall productivity. Consequently, firms’ motivation for tax avoidance decreases, confirming the existence of the “factor substitution effect” “innovation capability effect” and “resource allocation effect”. (3) Heterogeneity analysis demonstrates that the inhibitory effect of population aging on tax avoidance is more pronounced in labor-intensive firms, lower financing capabilities, weaker financial conditions, and firms operating in regions with a weaker external financial environment.

### 6.2 Research insights

Based on the research findings of this study, the following insights can be derived:

Firstly, at the conceptual level, it is crucial to have a correct understanding of population aging as a long-term trend in most developed and developing countries worldwide. It is important to objectively and dialectically recognize the profound and complex impacts of population aging on various resource factors of the economy and businesses. Population aging leads to a continuous reduction in labor supply, negatively affecting firm development and economic growth. However, declining fertility rates and smaller family sizes contribute to the accumulation of human capital and the improvement of labor supply quality, which in turn promotes research and development (R&D) innovation and enhances overall productivity. Thus, population aging has both positive and negative implications for firm development and economic growth.

Secondly, at the government level, corporate income tax plays a significant role in national fiscal revenue. This study demonstrates that although population aging has both positive and negative effects on firm resource factors, it ultimately has a positive impact on firms’ tax avoidance decisions and national fiscal outcomes. Governments should actively introduce policies that promote business innovation and development, driven by considerations of economic growth or national fiscal concerns. These policies could include measures to mitigate the decline in the labor force size, promote human capital accumulation and improvement, enhance labor force quality, facilitate business automation, digitization, and intelligence, and support and foster R&D innovation. Such policies not only enhance firms’ core competitiveness but also contribute to the growth of national fiscal revenue.

Thirdly, at the firm level, as important market participants, firms’ decision-making is influenced by the economic and social environment and its changes. Firms should have a comprehensive and deep understanding of the direct impact of population aging on their labor resources and the indirect impact on other resource factors such as capital, technology, and overall productivity. To safeguard long-term business interests, firms should make financial decisions that prioritize increased investment in fixed assets, accelerate business capitalization and digitization, enhance R&D innovation capabilities and levels, promote technological progress, improve overall productivity, fundamentally enhance competitiveness, and actively address the negative impacts of population aging.
